# Supplementary material for: Identification of photoperiod-induced specific miRNAs in the adrenal glands of Sunite sheep (Ovis aries)
Source: Front Vet Sci. 2022 Jul 22;9:888207. doi: 10.3389/fvets.2022.888207 (PMC9354845; doi:10.3389/fvets.2022.888207)
Supplement: Supplementary file 1 [file Data_Sheet_1.ZIP › Supplementary file/miRDeep2 file.zip!/miRDeep2 file/Novel_Structure/SP42A3/Novel_207.pdf]

Provisional ID	:	NC_019484.2_32033
Score total	:	14.1
Score for star read(s)	:	3.9
Score for read counts	:	6.8
Score for mfe	:	1.9
Score for randfold	:	1.6
Score for cons. seed	:	
Total read count	:	25
Mature read count	:	23
Loop read count	:	0
Star read count	:	2

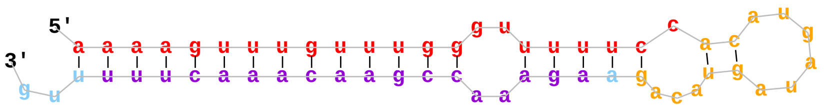

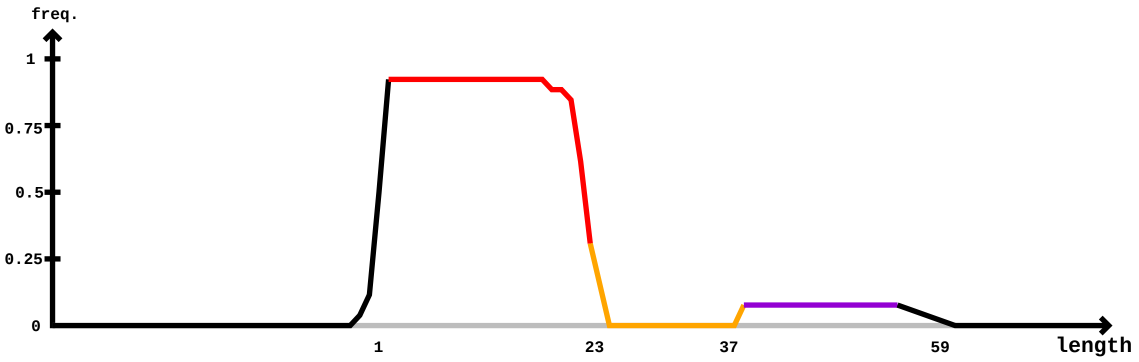


```
                                   Mature                         Star
5'- gggugaggaacucgguacuauugggguuggcaaaaaaguuuguuuggguuuuuccacaugauaguacagaagaaaccgaacaaacuuuuuggcuaauccaauagcagc   -3'    obs
    gggugaggaacucgguacuauugggguuggcaaaaaaguuuguuuggguuuuuccacaugauaguacagaagaaaccgaacaaacuuuuuggcuaauccaauagcagc          exp
    ((((.....))))....((((((((.(((((.(((((((((((((((..(((((.((......))...)))))..))))))))))))))).))))))))))))))....   reads    mm    sample
    ..............................Aaaaaaaguuuguuuggguu..........................................................   1        1     seq
    ...............................Caaaaaguuuguuuggguuuuu.......................................................   2        1     seq
    ................................aaaaaguuuguuuggguuuC........................................................   1        1     seq
    ................................aaaaaguuuguuCggguuuuu.......................................................   1        1     seq
    ................................aaaaaguuuguuuggguuuCu.......................................................   1        1     seq
    ................................aaaaaguuuguuuggguuuuuc......................................................   4        0     seq
    ................................aaaaaguuuguuGggguuuuuc......................................................   1        1     seq
    ................................aaaaUguuuguuuggguuuuuc......................................................   1        1     seq
    ................................aaaaaguuuguuuggguuuuucU.....................................................   1        1     seq
    .................................aaaaguuuguuuggguuuCu.......................................................   1        1     seq
    .................................aaaGguuuguuuggguuuuu.......................................................   1        1     seq
    .................................aaaUguuuguuuggguuuuuc......................................................   1        1     seq
    .................................Gaaaguuuguuuggguuuuuc......................................................   1        1     seq
    .................................Gaaaguuuguuuggguuuuucc.....................................................   3        1     seq
    .................................aaaaguuuguuuggguuuuucc.....................................................   4        0     seq
    ......................................................................agaaaccgaacaaacuuu....................   2        0     seq
```
